# Spatial analyses of threats to ecosystem service hotspots in Greater Durban, South Africa

**DOI:** 10.7717/peerj.5723

**Published:** 2018-10-26

**Authors:** Rashieda Davids, Mathieu Rouget, Richard Boon, Debra Roberts

**Affiliations:** 1 School of Agricultural, Earth and Environmental Sciences, University of KwaZulu-Natal, Pietermaritzburg, South Africa; 2 UMR PVBMT, CIRAD, Saint-Pierre, Reunion Island; 3 Environmental Planning and Climate Protection Department, eThekwini Municipality, Durban, South Africa; 4 School of Life Sciences, University of KwaZulu-Natal, Durban, KwaZulu-Natal, South Africa; 5 Sustainable and Resilient City Initiatives Unit, eThekwini Municipality, Durban, South Africa

**Keywords:** Ecosystem services, Urban planning, South Africa, Durban, Hotspots

## Abstract

**Background:**

Population growth at all scales and rapid rates of urbanization, particularly in the global South, are placing increasing pressure on ecosystems and their ability to provide services essential for human well-being. The spatial consideration of threats to ecosystem services related to changes in land use is necessary in order to avoid undue impacts on society due to the loss or reduced supply of ecosystem services. This study assesses the potential threats of land use change from strategic and local development proposals to ecosystem services in the city of Durban.

**Methods:**

We analysed the spatial relationship between five categories of ecosystem service hotspots (carbon storage, water yield, sediment retention, nutrient retention and flood attenuation) and urban land use change related to selected strategic planning proposals, development proposals and sand-mining applications in Durban, South Africa (eThekwini Municipality) with a view to determining the consequences for progress towards a more sustainable development path in the city. We identified the potential levels of threat related to habitat destruction or transformation for the five categories of ecosystem services and a subset of 13 ecosystem service hotspots, using GIS spatial analysis tools.

**Results:**

The results show that on average, should Durban’s strategic development plans be realised, approximately 42% loss of ecosystem service hotspots is expected in the two municipal town-planning regions assessed. With respect to development applications between 2009 and 2012, approximately 36% of all environmental impact assessments and 84% of sand mining applications occurred within ecosystem service hotspots within Durban.

**Discussion:**

The findings highlight the tension between short-term development pressures and longer-term sustainability goals and confirm that current planning and development proposals pose a threat to ecosystems and their ability to deliver services that support human well-being in Durban. We suggest practical solutions to include ecosystem services into local government decision-making.

## Introduction

Population growth at all scales and rapid rates of urbanization, particularly in the global South, are placing increasing pressure on ecosystems and their ability to provide services essential for human well-being (Steffen et al., 2015; United Nations, Department of Economic and Social Affairs (UN DESA), 2015). Ecosystem services are the life sustaining benefits that humans derive from natural ecosystems (MEA, 2005), more recently referred to as ‘nature’s contributions to people (NCP)’, which includes positive and negative contributions of nature to human quality of life (Diaz et al., 2018). Ecosystem services include regulating services (or regulation NCP) such as climate, water, air, hazard and disease regulation; provisioning services (or material NCP) such as healthy food supply, materials and medicines and cultural services (or non-material NCP) including education, inspiration, recreation, physical and psychological experiences (Costanza et al., 1997; Daily, 1999; MEA, 2005; TEEB, 2011; Diaz et al., 2018).

Anthropogenic environmental pressures including the conversion of natural ecosystems into urban areas has contributed impacts on global biodiversity hotspots such as species decline (Rockström et al., 2009; Seto, Güneralp & Hutyra, 2012), environmental degradation and climate change (Steffen et al., 2015). The negative consequences for human well-being due to impacts on ecosystems and ecosystems services (Larigauderie et al., 2012; MEA, 2005; McGranahan et al., 2005) will be compounded by risks associated with often abrupt, irreversible and non-linear ecosystem changes in response to disturbance regimes (Rockström & Karlberg, 2010). Increasing populations in cities also mean greater demands for the life supporting ecosystem services provided by natural systems. In cities of the global South, these demands are exacerbated by poverty and direct dependence on ecosystem services for livelihoods and well-being (Shackleton & Shackleton, 2004; Stoian, 2005; Davenport, Shackleton & Gambiza, 2012).

Despite their critical importance to human welfare, a consideration of ecosystem services is insufficiently integrated into landscape planning and management processes and decision making (Daily et al., 2009; De Groot et al., 2010; Nemec & Raudsepp-Hearne, 2013). The neglect of ecosystem services in decision making can be attributed to the complexity of the ecosystem processes involved, including that services are often produced at some distance from urban beneficiaries (Baró et al., 2016); they rarely conform to property or administrative boundaries; public agencies find it difficult to manage and regulate them (McGranahan et al., 2005) and the fact that those most affected by the loss of ecosystem services are the urban poor, who are the least economically and politically influential (Shackleton & Shackleton, 2004; Stoian, 2005; Davenport, Shackleton & Gambiza, 2012).

The ecosystem services approach is, however, increasingly seen as a tool that allows for entry into a broader set of social and political processes, with an expectation that this holistic approach could be integrated at all governance levels and provide a basis for policy design (Díaz et al., 2015; Primmer et al., 2015). New research is investigating various modes of governance, that consider inputs and decision-making from a range of stakeholders, for all elements of ecosystem services, from ecosystem structure, functions and services, to benefits and values (Primmer et al., 2015; Potschin & Haines-Young, 2011). This is most evident through the establishment of the Intergovernmental Science-Policy Platform on Biodiversity and Ecosystem Services (IPBES)—the intergovernmental body which assesses the state of biodiversity and of the ecosystem services it provides to society.

The 2030 Agenda for Sustainable Development recognizes the need to reduce the impact of urban development on life-supporting natural systems with the inclusion of Sustainable Development Goal (SDG) 11 focused on creating inclusive, safe, resilient and sustainable cities (United Nations Development Programme, 2016). Rockström & Sukhdev (2017) highlight that natural capital and ecosystem services are foundational in achieving the SDGs and their associated targets, given that sustainable social and economic development is only possible if it occurs within the limits of the environment. To this end, the targets associated with SDG 11 recognize the need for the protection of natural heritage, the reduction of environmental impacts and for development planning that is strengthened though positive economic, social and environmental links between urban, peri-urban and rural areas (United Nations, 2015a; United Nations, 2015b).

The city of Durban in South Africa has shown commitment to this understanding of sustainable development (and more recently the SDGs) by virtue of its long history of biodiversity and ecosystem services planning and management that culminated in the establishment of the Durban Metropolitan Open Space System (D’MOSS) (McLean et al., 2016). Numerous threats to biodiversity and the associated delivery of ecosystem services, however, still remain within the city including habitat destruction and fragmentation—a minimum of 53% of Durban’s natural areas have already been transformed by human activities (eThekwini Municipality, 2012a)—and climate change (Carmin, Anguelovski & Roberts, 2012).

City wide strategic planning and localized development proposals are considered to be significant drivers of land use change in Durban. Ecosystem services are extremely vulnerable to human induced land use change impacts (Arunyawat & Shrestha, 2016). The spatial consideration of threats related to changes in land use with respect to ecosystem services is necessary in order to help avoid inappropriate land uses that could reduce the supply of ecosystem services (Arunyawat & Shrestha, 2016) and could greatly improve the selection of areas where the best conservation outcomes could be achieved (Evans et al., 2011). Conservation planning is proposed as a tool for responding to threats associated with land transformation, which in addition to habitat loss through urbanisation and the associated expansion of infrastructure, includes extractive land uses (agriculture, forestry, mining, grazing) and the spread of invasive alien species (Wilson et al., 2004).

In this study, we use Durban as a case study to assess potential levels of threat to five categories of ecosystem service hotspots (Davids et al., 2016), namely, carbon storage, water yield, flood attenuation, sediment retention and nutrient retention, related to habitat destruction or transformation, which may result from the implementation of strategic development proposals (as outlined in the Strategic Development Plans for the Outer west and North regions of the city), local development proposals (as identified through the associated environmental impact assessments) and sand mining proposals, using spatial analysis tools in ArcGIS.

## Methods

### Study area

Durban is administered by a local government authority known as eThekwini Municipality and is situated in the province of KwaZulu-Natal, South Africa. In 2015 the municipal area of Durban was approximately 229,193 ha in extent (1.4% of the province) with a population 3.55 million (Davids et al., 2016). Durban’s coastline is 98 km long, and is dissected by the rivers of 18 major water catchments and 16 estuaries. Furthermore, Durban is located within the Maputaland-Pondoland-Albany global biodiversity hotspot, rated as such because of its high levels of plant endemism and habitat loss (Mittermeier et al., 2004).

Durban contains urban, peri-urban and rural environments, with approximately two-thirds of the municipal area being rural or semi-rural, where a large proportion of local inhabitants are indigent and directly reliant on ecosystem services for basic needs (Roberts & O’Donoghue, 2013; Sutherland et al., 2014). Social challenges in Durban include high levels of poverty; many densely populated informal settlements; unequal basic service delivery; high rates of urbanisation and dual governance arrangements, whereby eThekwini Municipality jointly administers communal land in the rural northwest and southwest areas of the municipality with the Ingonyama Trust Board (under the jurisdiction of traditional councils) and provincial government (McLean et al., 2016; Sutherland et al., 2014; Davids et al., 2016). These challenges have often meant that socio-economic development priorities have taken preference over environmental and biodiversity concerns (Roberts, 2008).

The Durban Metropolitan Open Space System (D’MOSS) is formally included in eThekwini Municipality’s hierarchy of spatial plans and policies, and serves as a legal instrument for environmental protection through development and use restrictions. D’MOSS comprises natural areas of high biodiversity value within a series of interconnected open spaces, aimed at protecting globally significant biodiversity and the supply of ecosystem services (Roberts & O’Donoghue, 2013; Davids et al., 2016).

### Overview of South African spatial planning products considered in this study

In terms of the Constitution of the Republic of South Africa, 1996 (as amended), spatial planning in South Africa is the responsibility of all three spheres of government, local, provincial and national. The national Spatial Planning and Land-use Management Act (SPLUMA), 2013 (Act No. 16 of 2013) provides the framework for all land use management and spatial planning legislation in South Africa. This Act has numerous aims, including the regulation of planning procedures and decision making; addressing spatial imbalances that resulted from the apartheid era and ensuring integration of sustainable development principles in land use planning and regulatory tools.

In terms of SPLUMA, all spheres of government must prepare spatial plans, however, land use management is the responsibility of municipalities, in participation with local traditional councils. In Durban, the traditional councils administer communal lands totalling ±82,266 ha (35.8%) (Davids et al., 2016). SPLUMA and the Municipal Systems Act, 2000 (Act No. 32 of 2000) require that local municipalities develop land use management systems, including the implementation of Integrated Development Plans (IDPs) and municipal spatial development frameworks (SDFs) and associated land use guidelines. EThekwini Municipality’s IDP (2012/2013) considered in this study, was developed with the aim to address key strategic issues identified in the National Spatial Vision and Provincial Growth and Development Strategy, including job creation; reversing the effects of apartheid; access to quality of education, healthcare and social protection; and the transition to a low carbon economy (eThekwini Municipality, 2012b). The IDP states that in order to suitably manage development and minimise impacts on the natural environment and associated ecosystem services, spatial planning must be enhanced and better aligned with the strategic development plans of the Municipality (eThekwini Municipality, 2012b).

## Data

### Ecosystem service hotspots

The classification, mapping and spatial prioritisation of ecosystem services has received considerable attention (Egoh et al., 2008; Schröter & Remme, 2016; Cimon-Morin, Darveau & Poulin, 2013; Schröter et al., 2017). Among other approaches, the delineation of ‘ecosystem service hotspots’ has been used to spatially prioritise ecosystem services in conservation planning (Cimon-Morin, Darveau & Poulin, 2013). Ecosystem service hotspots either refer to areas of high ecosystem service values of one service, or areas with a combination of multiple services (Schröter & Remme, 2016).

**Figure 1:**
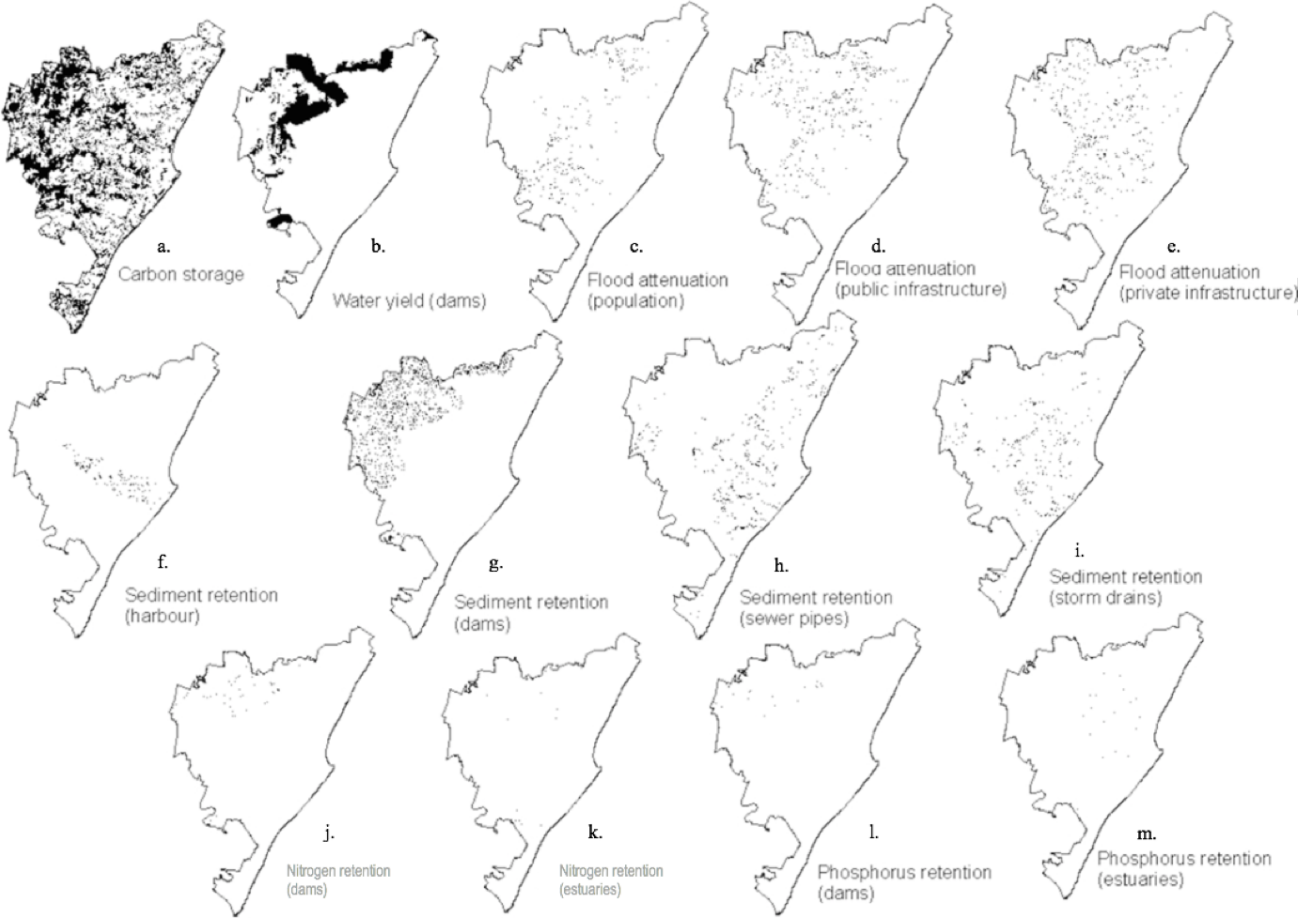
Hotspots for 13 ecosystem services in the eThekwini Municipal Area (from Davids et al., 2016). (A) Carbon storage. (B) Water yield. (C) Flood attenuation (population). (D) Flood attenuation (public infrastructure). (E) Flood attenuation to (private infrastructure). (F) Sediment retention (harbour). (G) Sediment retention (dams). (H) Sediment retention (sewer pipes). (I) Sediment retention (storm drains). (J) Nitrogen retention (dams). (K) Nitrogen retention (estuaries). (L) Phosphorus retention (dams). (M) Phosphorus retention (estuaries).

Davids et al. (2016) identified hotspots for 13 ecosystem services in Durban, grouped into five categories (Table 1). The 13 ecosystem services are: carbon storage, water yield (to dams), four sediment retention services (preventing sedimentation of dams, stormwater and sewer pipes and the harbour), three flood attenuation services (relevant to the population, private and public infrastructure) and four nutrient retention services (phosphorus and nitrogen relative to both dams and estuaries) (Table 1; Fig. 1). The original ecosystem service maps for these 13 services were commissioned by the municipality’s Environmental Planning and Climate Protection Department in 2012 and were derived using the InVEST tool, developed by the Natural Capital Project (Tallis & Polasky, 2009). InVEST allows for priority ecosystem service areas to be identified based on a number of factors that either contribute, to or impact on selected ecosystem services (J Glenday, 2012, unpublished data submitted to the eThekwini Municipality). ArcGIS 9.3 was used to estimate the ecosystem service provision based on biophysical properties, land over and location relative to downstream populations, property and infrastructure, along land surface and river channels flow paths (J Glenday, 2012, unpublished data submitted to the eThekwini Municipality). These ecosystem services were linked to a standard 86.9 m resolution and scaled to values ranging from 0 to 100, to indicate relative service provision compared to the maximum found in the study area.

**Table 1:** Ecosystem functions and their contribution to the services (J Glenday, 2012, unpublished data submitted to the eThekwini Municipality).

Five ecosystem service categories	Sub-set of ecosystem service categories
*Carbon storage*	1. Mitigating global climate change
*Water yield*	2. Providing water supply to dams
*Sediment retention*	3. Reducing need for harbour dredging
	4. Reducing loss of dam capacity to sedimentation
	5. Reducing sewer pipe maintenance due to sedimentation
	6. Reducing stormwater pipe and culvert maintenance due to sedimentation
*Nutrient retention (nitrogen and phosphorus in runoff)*	7. Nitrogen retention for improving water quality in dams (reducing harmful algal blooms, filtration needed for domestic water supplies, alien water plant proliferation, and dam system maintenance)
	8. Phosphorus retention for improving water quality in dams
	9. Nitrogen retention for improving water quality in estuaries for fisheries and recreation
	10. Phosphorus retention for improving water quality in estuaries for fisheries and recreation
*Flood attenuation*	11. Reducing negative flood impacts on populations living in floodplain areas (loss of life, loss of quality of life)
	12. Reducing flood damage to private property
	13. Reducing flood damage to public infrastructure

**Figure 2:**
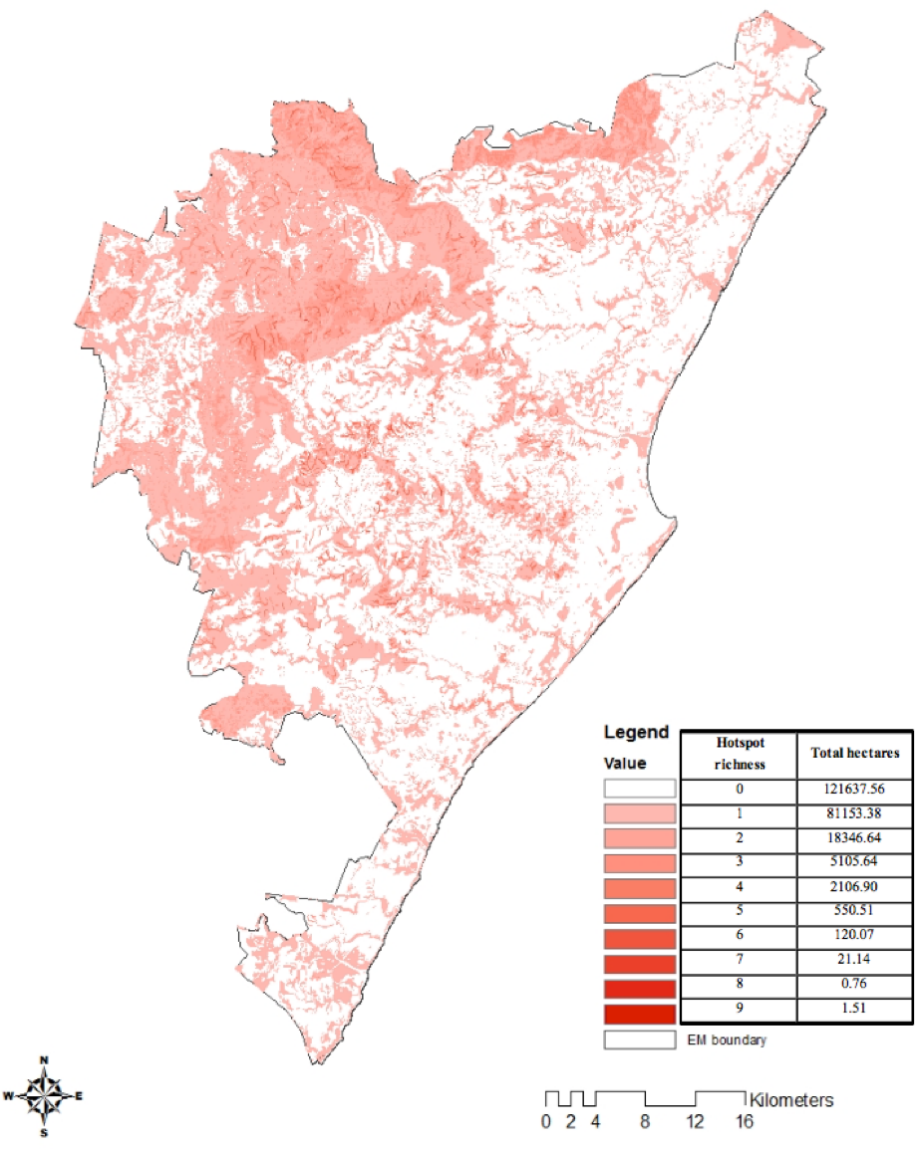
Ecosystem service hotspot richness map.

Ecosystem service hotspots were defined by Davids et al. (2016) as the top 50% of ecosystem service provisioning areas (Fig. 1). These ecosystem service hotspots represent the provisioning areas of a particular service that had values greater than the median value (Davids et al., 2016). The median value was used to reduce the importance of outliers present (Press et al., 1992) in the range of ecosystem service values and to avoid the exclusion of large areas providing good ecosystem services (Davids et al., 2016). Approximately 35% of Durban was considered a hotspot for at least one of the 13 ecosystem services assessed (Fig. 2). Majority of the 13 ecosystem service hotspots identified by Davids et al. (2016) were found to be outside of environmental management areas and threatened by habitat transformation, rapid densification, invasive alien plant invasions and pollution, with only half of ecosystem service hotspots located within D’MOSS. The hotspot richness map (Fig. 2) was developed using overlay analysis, which merged the distributions of 13 ecosystem services provisioning maps into a raster grid (with a presence value of 1 for each map) to show the total number of ecosystem services produced in a particular location from a minimum of 0, to a maximum of 13 (Schröter & Remme, 2016; Davids et al., 2016).

### Spatial development framework

The SDF forms part of a hierarchy of integrated plans that make up the Land Use Management System currently being established to manage land use and development within Durban (eThekwini Municipality, 2012b). The intention of the SDF is to guide all decisions related to the use, development and planning of land within Durban, providing a strategic framework that spatially indicates how the implementation of the city’s IDP should occur. The SDF is a five-year plan, which is revised annually in line with the IDP. It provides strategic multi-sectoral planning guidance relative to development priorities, transport planning, bulk infrastructure and environmental directives, and acts as a guide to more detailed Local Area Plans, Functional Area Plans, detailed Precinct Plans and Land Use Schemes. This study assesses the proposed changes to land use that could occur through the implementation of the eThekwini Municipality Spatial Development Plans against “the five categories of ecosystem service hotspots and the subset of 13 services.”

The terminologies for land uses in the SDPs were not standardized and categories of land use were grouped as follows: natural areas for environmental protection, nature-based recreation and tourism, amenity, agricultural use and infrastructure.

### Spatial planning regions

**Figure 3:**
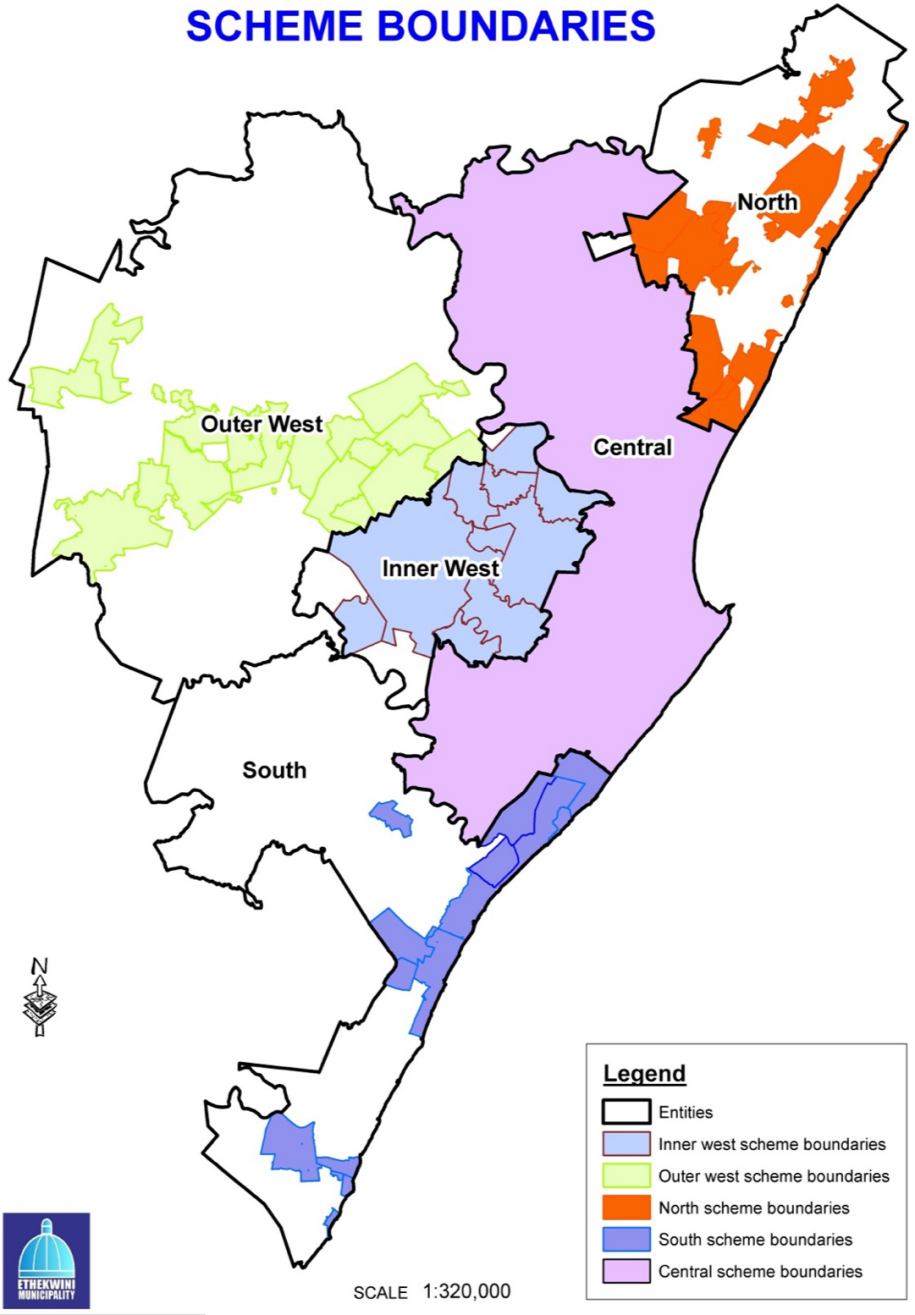
eThekwini Municipality Spatial Planning Regions and areas within these covered by town planning schemes.

Durban’s municipal area is divided into four major planning regions, namely, North, South, Central (including Inner West) and Outer West (Fig. 3). Within Durban, only 36% of land is covered by town planning schemes and formally administered by the municipality, approximately 38% is communal land jointly administered by the traditional councils, and the municipality and 26% is peri-urban and jointly administered by local and provincial government (McLean et al., 2016). A process is currently underway to incorporate traditional communal lands and agricultural areas into town planning schemes (McLean et al., 2016).

Under the SDF, four individual Spatial Development Plans (SDP) have been prepared and were adopted in November 2009 and revised in November 2010 and 2011 (eThekwini Municipality, 2012b). Two SDPs (North and Outer West) were selected for use in this study, due to their contrasting general land uses, that is one being more rural in the case of the Outer West SDP and the other more urban in the case of the North SDP.

### Historical development applications

Historical development applications assessed in this study include all environmental impact assessments and sand mining applications that were submitted to the Environmental Planning and Climate Protection Department of eThekwini Municipality, between 2009 and 2012. The applications were linked to a point file in GIS, thus the points used in the analyses are merely an indication of where transformation of land may be expected, in the event that applications were successful.

**Table 2:** Ecosystem service Strategic Development Plan analyses combinations and summary.

	Strategic development plans (two selected planning regions)	
	**North region**	**Outer West region**
Allocation of future land uses to the 13 Individual ecosystem services areas	–54% of all ES areas in environmental land use: carbon storage (62%), flood attenuation public and private infrastructure (61% each) and phosphorus retention dams and estuaries (75% each)	–62% of all ES areas in environmental land use: carbon storage (82%), flood attenuation public and private infrastructure (77% each), sediment retention harbor (74%)
	–40% of urban residential in sediment retention storm drains and sewer pipes and 38% urban residential in nitrogen retention estuaries	–51% of water yield, 47% of nitrogen retention dams and 42% nitrogen retention estuaries in rural residential & tourism
	–26% agriculture to fall within water yield, 30% in sediment retention dams and 32% in nitrogen retention dams	–33% of phosphorus retention estuaries in urban residential
Allocation of future land use to the 5 ecosystem service categories (carbon storage, water yield, sediment retention, nutrient retention & flood attenuation)	–60% at risk of transformation	–47% at risk of transformation
	–38% of all ES areas in urban and rural residential land use: carbon storage (21%), water yield (34%), sediment retention (36%), nutrient retention (71%), flood attenuation (27%)	–33% of all ES areas in urban and rural residential land uses: carbon storage (10%), water yield (53%), sediment retention (34%), nutrient retention (51%), flood attenuation (17%)
	–18% of all ES areas in agricultural land use: carbon storage (15%), water yield (34%), sediment retention (24%), flood attenuation (15%)	–2% of all ES areas in agricultural land use
	–39% of all ES areas in environmental land use	–63% of all ES areas in environmental land uses: carbon storage (86%), water yield (36%), sediment retention (62%), nutrient retention (47%), flood attenuation (82%)
	carbon storage (60%), water yield (26%), sediment retention (33%), nutrient retention dams (21%), flood attenuation (54%)	

## Analyses

**Figure 4:**
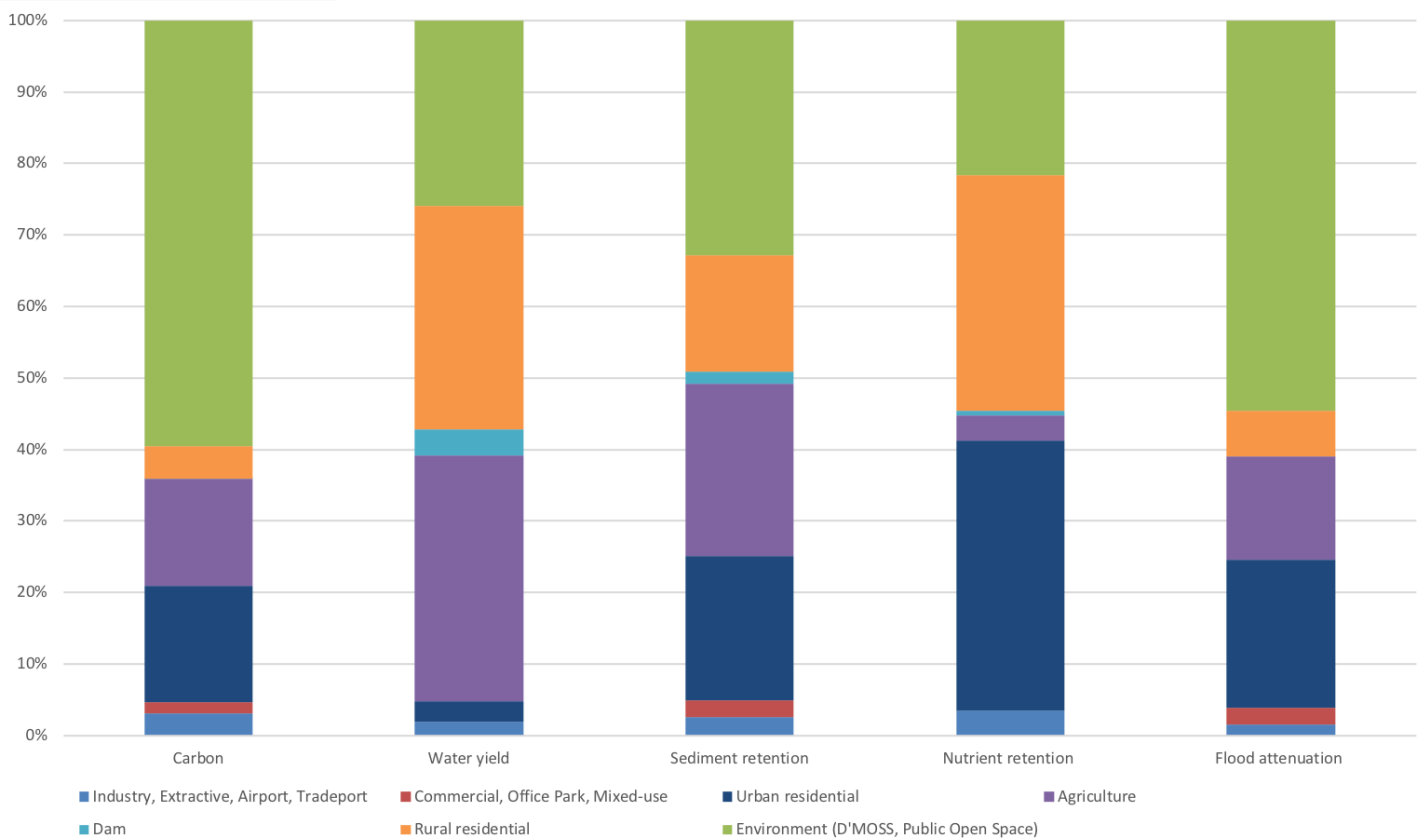
Proportions of proposed land use relative to ecosystem function hotspots in the Northern Planning Region (%).

**Figure 5:**
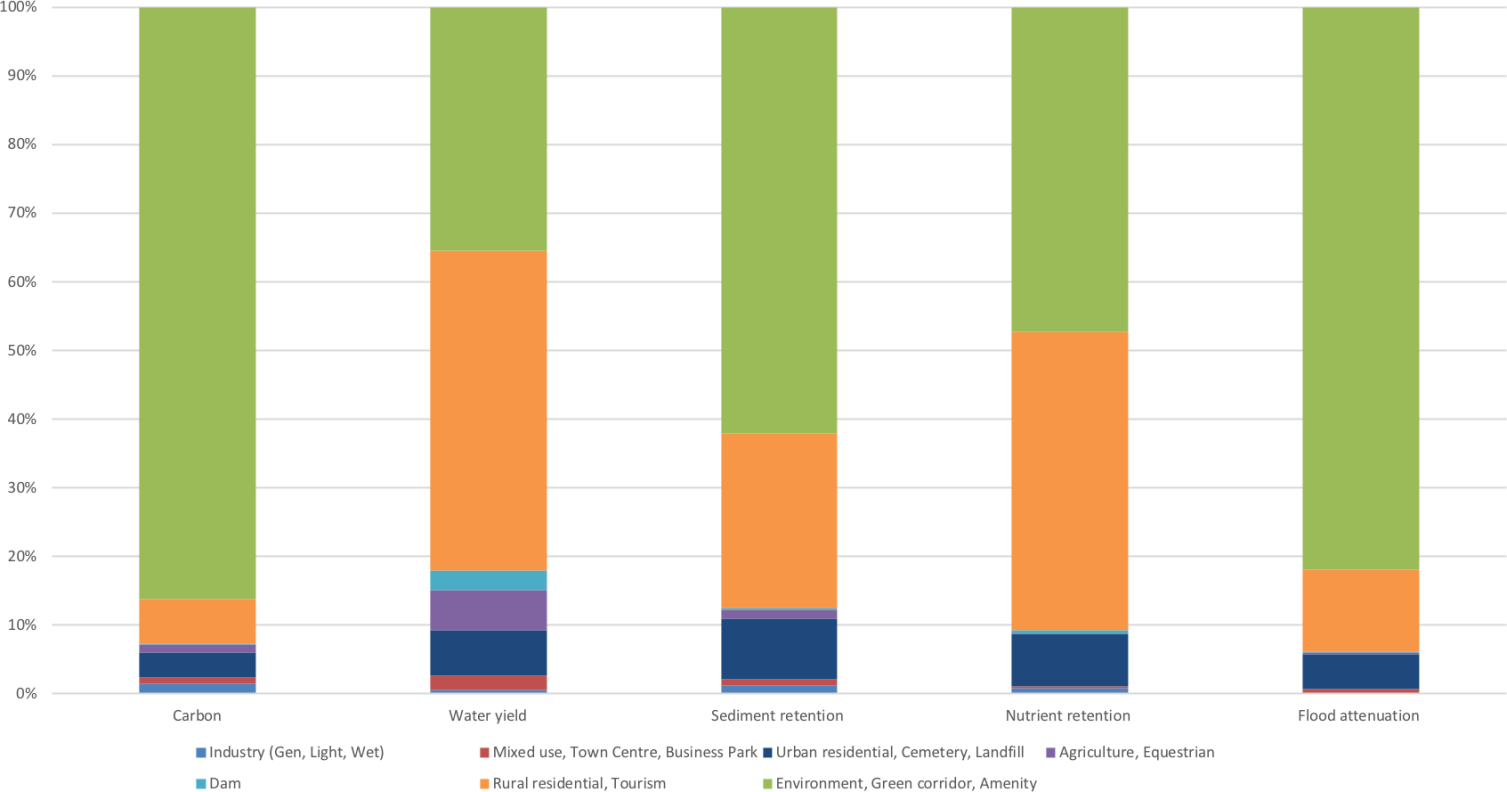
Proportions of proposed land use relative to ecosystem function hotspots in the Outer West Planning Region (%).

We analysed potential transformation of land supplying ecosystem services for various ecosystem service-threat combinations, as shown in Tables 2 and 3. In addition to assessing individual services, we also grouped the 13 ecosystem services hotspots into the five main ecosystem service categories, namely, carbon storage, water yield, sediment retention, nutrient retention and flood attenuation (Tables 1 and 2; Figs. 4 and 5) and analysed these services against potential transformation relative to strategic (Table 2) and local development threats (Table 3) in ArcGIS 10.1. For this analysis, we generated five ecosystem services maps (one for each category) by aggregating all ecosystem services maps per category based on the maximum value per cell.

**Table 3:** Ecosystem service Strategic Development Plan analyses combinations and summary.

	Development proposals (entire eThekwini Municipal Area)	
	**Environmental Impact Assessment (EIA) sites**	**Sand mining sites**
13 Individual ecosystem services and their allocation to future land use	–36% of all EIAs in ecosystem service hotspots	–84% of sand mining proposals in ecosystem service hotspots
	–Of all EIAs in hotspots: 68% made in carbon storage hotspots, 8% in sediment retention hotspots, 7% in water yield hotspots	–Of all sand mining applications in hotspots: 86% in carbon storage hotspots and 7% in water yield hotspots
Ecosystem service hotspot richness relative to proposed development sites	–84% in areas providing only one service	–93% in areas providing one service
	–9% in areas providing two services	–4% within areas with two services
	–5% within areas providing three services	–2% within areas providing three services and
	–2% in areas providing four services	–1% in areas providing four services
	–No application in hotspot richness of 5 or more	–No application in hotspot richness of 5 or more

To analyse the threat of transformation of land supplying ecosystem services related to the implementation of the SDPs in the North and Outer West Regions, we first grouped the planned land uses in each region in terms of their compatibility with ecosystem services. The compatibility of proposed land uses to the continued supply of ecosystem services was determined based on the potential of that land-use to maintain ecosystems in their natural or semi-natural state (rated as ‘compatible’) or where land is expected to be significantly modified (e.g., cultivated or urban), with natural areas largely transformed (rated as ‘incompatible’). Although we acknowledged that significantly modified areas are able to produce some degree of ecosystem services (Colding, Lundberg & Folke, 2006), for this study we assumed service values for these areas to be lost when transformed to ‘incompatible’ land uses (Reyers et al., 2009).

Compatible land uses include D’MOSS, environment, public open space, green corridor, amenity, dam, tourism (Figs. 4 and 5; Appendices S1–S4). D’MOSS is included within the municipal town planning schemes as a controlled development layer (Roberts & O’Donoghue, 2013) and comprises protected areas, nature reserves and terrestrial, estuary and freshwater biodiversity areas, and environmental approval is required prior to any development taking place within designated D’MOSS areas, thus offering some form of protection from transformation (Davids et al., 2016). These biodiversity areas have been shown to support ecosystem service production (Davids et al., 2016; Chan et al., 2006; Egoh et al., 2009; Turner et al., 2007; Singh, 2002).

Incompatible uses include land that will be transformed from natural or semi-natural states and would result in reduced or lost productivity of biodiversity and associated the ecosystem services (De Groot et al., 2010; McGranahan et al., 2005), namely; agriculture, equestrian, urban and rural residential, rural cemetery, landfill, mixed use, town centre, business park, industry, extractive, airport and tradeport (constituting the support zone to airport-related activities including infrastructure, offices, warehouses and cargo terminal). We grouped agricultural land-use as being not compatible with ecosystem services, based on the understanding that increasing productivity generally results in a loss or decline in ecosystem services (Kareiva et al., 2007), whereby agriculture may result in negative impacts on biodiversity though direct modifications (removal or addition of biota: predators, pests and parasites of domestic species) and indirect modifications (changes to biogeochemical cycles, and changes to hydrological cycles); and changes to the habitats of native species (Baudron & Giller, 2014).

The percentage of ecosystem services provision areas that may be lost due to incompatible land-uses was calculated based on the results of raster calculator analyses of each ecosystem service hotspot with the various proposed land uses in the two SDP regions analysed.

Development and sand mining proposals pose potential threats to ecosystem services due to biodiversity impacts in the form of vegetation clearing and the removal of life-supporting topsoil (eThekwini Municipality, 2012a). To analyse the potential levels of threat to ecosystem services linked to development proposals, the locations of all environmental impact assessment and sand-mining applications were overlaid with each of the 13 ecosystem service hotspots, to assess the proportion of applications that were planned to occur in these hotspots. These locations were also overlaid with an ecosystem service hotspot richness map (Davids et al., 2016), to assess the proportion of applications that were planned to occur within areas that supply a combination of ecosystem services. The limitation here is that the positions alone do not provide a quantitative assessment of the extent of potential transformation of ecosystem service hotspots. However, this analysis provides an indication of the demand for transformation of natural areas providing ecosystem services related to the volume of applications made in ecosystem hotspots.

## Results

### Spatial development plans: outer west and north planning regions in relation to priority areas for the five categories of ecosystem services

The proposed implementation of the SDPs will affect ecosystem service provision in both regions. On average, 60% and 47% of ecosystem service hotspot areas in the North and Outer West respectively, are at risk of being degraded or lost, as they will fall outside of land uses specifically designated to remain natural, such as environment, D’MOSS, public open space, green corridor and amenity (Table 2; Fig. 4; Appendix S3, Fig. 5, Appendix  S4).

On average, approximately 39% for the North and 63% for the Outer West region is proposed to remain within areas designated for environmental purposes, compatible with ecosystem service conservation, namely, D’MOSS, Public Open Space or Amenity. The situation with water yield and nutrient retention to dams is dire with the proposed implementation of the SDP in these two planning regions, leading to about 26% and 21% respectively of the hotspot areas remaining within environmental areas in the North and 36% and 47% respectively in the Outer West. Sediment retention services are also poorly provided for in the North, with only 33% falling within an environmental land-use.

In both regions, the proposed residential land use in the SDPs overlaps with the highest proportions of ecosystem services hotspot areas, with approximately 33% allocated for combined rural and urban residential uses in the Outer West (Appendix S3) and 38% combined rural and urban residential uses in the North (Appendix S4). The other proposed land-uses that are not compatible with ecosystem services, namely industry, commercial, office park and mixed use comprise comparably nominal proportions at between 1.3% and 2.5% in the North and 0.85 and 1% in the Outer West.

The five categories of ecosystem services will be variably affected by the North SDP, with an average of 18% proposed to fall within agricultural areas, of which 15% are carbon storage areas, 24% sediment retention areas, 34% in water yield ecosystem service areas and 15% of flood attenuation.

### Development proposals within ecosystem service hotspots

Approximately 36% (*n* = 658) of all EIA applications within Durban between 2009 and 2012 fell within an ecosystem service hotspot, while 84% (*n* = 144) of sand mining applications were for locations within ecosystem service hotspots (Tables 3 and 4). The ability to store carbon could be impacted as the highest number of EIA and sand mining applications are noted within carbon storage hotspots (68% and 86% of applications respectively of the total applications made within ecosystem service hotspots, i.e., the 36%).

**Table 4:** EIA and sand mining within ecosystem service hotspots.

	No. of EIAs	% EIAs in ES area	No. of sand-mining applications	% Sand-mining in ES area
1. Durban				
Totals	658	36.3	144	84.0
2. Ecosystem services areas				
Carbon	164	68.6	105	86.8
Water yield	17	7.1	8	6.6
Flood attenuation Pop	3	1.3	–	–
Flood attenuation public infrastructure	2	0.8	1	0.8
Flood attenuation private infrastructure	5	2.1	1	0.8
Sediment retention-dams	5	2.1	3	2.5
Sediment retention-sewer pipes	19	7.9	2	1.7
Sediment retention-storm drains	16	6.7	1	0.8
Sediment retention harbour	7	2.9	–	–
Nitrogen retention-dams	–	–	–	–
Nitrogen retention-estuaries	–	–	–	–
Phosphorus retention-dams	–	–	–	–
Phosphorus retention-estuaries	1	0.4	–	–
Total	239	100	121	100

**Figure 6:**
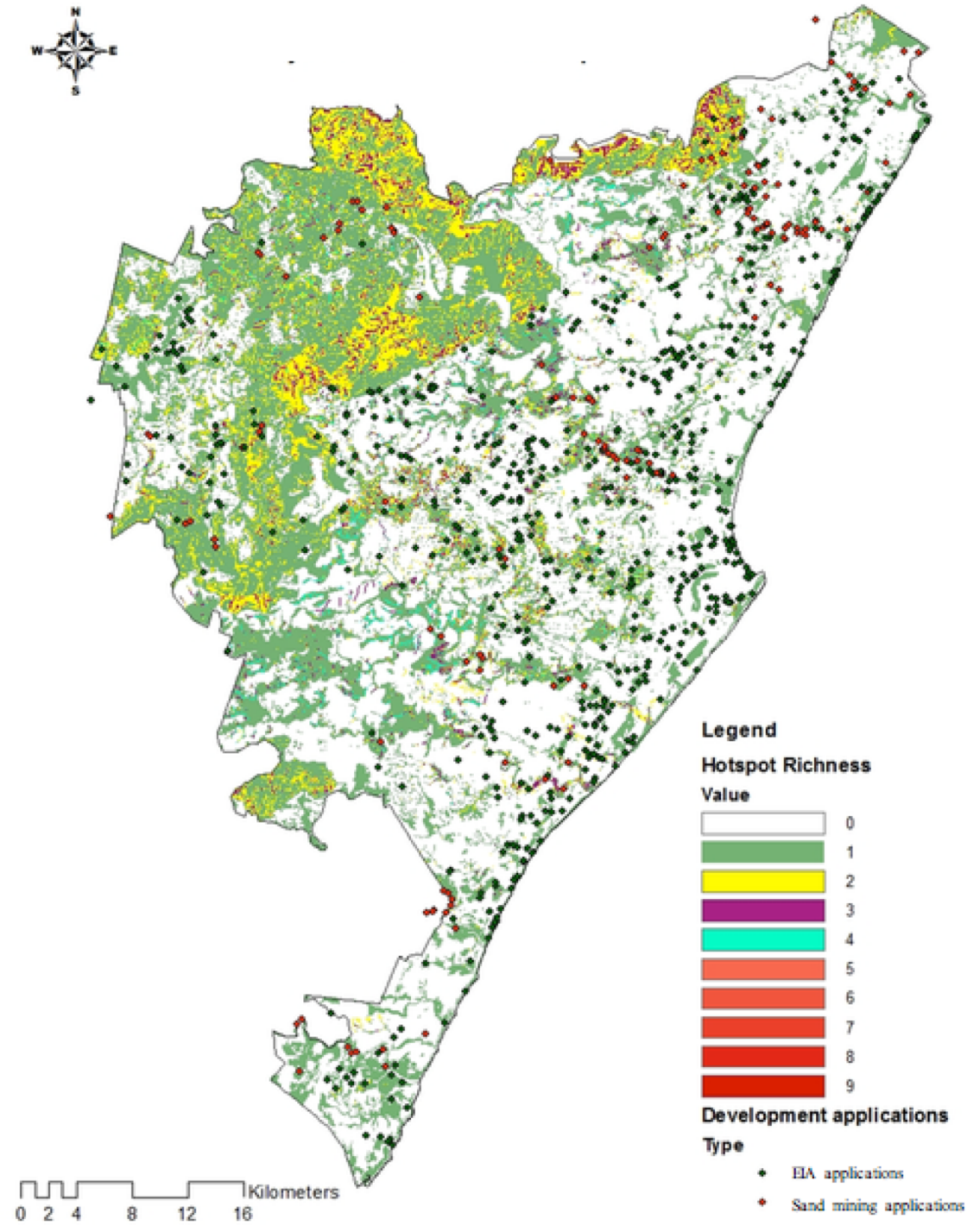
Development and sand mining applications relative to hotspot richness.

With respect to areas providing multiple ecosystem services (Fig. 6), the vast majority of EIAs occurred in areas that only produce one ecosystem service (84%), with approximately 9% within areas with two services, 5% within areas providing three services and 2% in areas providing four services. Similarly, for sand-mining applications, 93% of them occurred in areas that only produce one ecosystem service, approximately 4% within areas with two services, 2% within areas providing three services and 1% in areas providing four services.

No EIA or sand mining applications were made in areas with a hotspot richness of five or more services.

## Discussion

### Implications of threats to ecosystem services

The findings that the implementation of the SDPs might result in an average transformation or loss of 49% of ecosystem service hotspots, confirm habitat loss as a threat to the continued supply of ecosystem services within urban areas, and the need for effective municipal planning to reduce this threat (Davids et al., 2016; Daily, 2000; Driver, Cowling & Maze, 2003). These threats are emphasised by the finding that approximately 36% of EIA applications and 84% of sand mining applications made between 2009 and 2012, were located within ecosystem service hotspots.

Our study highlights the importance of the use of ecosystem service hotspots in conservation planning (Egoh et al., 2008; Schröter & Remme, 2016; Schröter et al., 2017; Cimon-Morin, Darveau & Poulin, 2013) as a potential tool to both avoid foreseeable threats to ecosystem services and human well-being, and to inform conservation and management to ensure the sustainability of ecosystem services (Davids et al., 2016; Schröter et al., 2017). The implications of the findings and options for management of the ecosystem service hotspots considered in this study, are discussed below.

The potential loss of carbon storage hotspots, as identified in this study, would be counterproductive to local government strategies to combat climate change. Anthropogenic changes have already resulted in the planetary boundaries for climate change and biodiversity loss being exceeded (Steffen et al., 2015; Rockström et al., 2009). The magnitude of change in supply of ecosystem services that were identified in this study, coupled with the inability of remaining natural areas to meet certain biodiversity targets in Durban (McLean et al., 2016), may push Durban into a state where critical environmental thresholds are crossed, and trigger further non-linear system changes (Steffen et al., 2015; Rockström & Karlberg, 2010). Although climate change is recognized as a global phenomenon, responses to climate change are being implemented at the local level, raising the importance of local governance in reducing climate-induced risks on communities (Williams et al., 2018; Surminski et al., 2017).

Over 50% of disaster-related fatalities are attributed to flood events (Wehn et al., 2015) and a third of economic losses are linked to climate induced risks, with the risk of flooding predicted to increase with global warming (Hirabayashi et al., 2013). The proposed increase in developed ‘incompatible’ land uses will result in increased runoff, and coupled with the potential loss of flood attenuation ecosystem services, may exacerbate flooding impacts with consequences for vulnerable indigent communities, particularly those living in informal settlements in flood prone areas (Arunyawat & Shrestha, 2016; Im et al., 2009; Du et al., 2012; Williams et al., 2018). This may also result economic costs related to loss or damage to public and private infrastructure.

The potential impacts on sediment retention services could result in soil erosion and sedimentation of rivers and dams, threatening water quality and aquatic food sources. In addition, the loss of sediment retention services could lead to blockages of stormwater infrastructure, with resultant economic costs related to the maintenance of the same. About a third of sediment retention services are planned to be replaced by agricultural land uses in both regions. In light of potential impacts associated with agricultural practices including increased runoff and soil erosion and resultant effects on water quality (Arunyawat & Shrestha, 2016; Kareiva et al., 2007; Baudron & Giller, 2014), sustainable agricultural practices that limit unwanted impacts on ecosystems and their services to human development are needed (Rockström & Karlberg, 2010).

The substantial amount of nutrient retention hotspots that would be impacted or lost by transformation to rural residential land use may result in increased nutrient loading in estuaries and dams and infiltration of nitrogen into groundwater, impacting on aquatic food sources and drinking water and posing a threat to human health (Cirone & Duncan, 2000). These impacts would also have cost implications for the municipality related to the treatment of water for supply of potable water to residents. In order to mitigate costs and enhance the quality of water supplied, interventions to enhance natural purification of water through wetlands and other habitats and mitigation of water quality degradation due to agriculture and urban development should be considered (Berka, Schreier & Hall, 2001; Houlahan & Findlay, 2004; Verhoeven et al., 2006).

The transformation of water yield service areas to agricultural areas and residential and uses will affect water resources. This impact would depend on a range of factors including the types of vegetation before and after transformation (e.g., affecting evapotranspiration rates and moisture transfer rates to soil), whether the change is permanent or temporary and the proposed land use practices related to, for example, irrigation and fertilization applications (Scanlon et al., 2007). Increases in rain-fed cultivation, increases in built-up areas and decreases in forest cover have been shown in increase water yield and decrease water quality (Arunyawat & Shrestha, 2016; Scanlon et al., 2007). However, increases in surface- or ground-water irrigated agriculture would decrease both water quantity and quality (Scanlon et al., 2007). The condition of natural areas within catchments that supply surface water through runoff must be maintained to ensure yields of high water quality, reduced nutrient loss and reduced soil erosion (Scanlon et al., 2007; Egoh et al., 2008).

### Suggestions for improved planning and management of ecosystem services

The inclusion of some ecosystem services into Durban spatial plans such as D’MOSS seems to be partially effective. Some of the reasons include that such planning allocations do not necessarily preclude development, nor do they ensure management of biodiversity in these areas. The challenge remains to efficiently govern ecosystem services in a systematic way. The effective management of ecosystem services would involve the structured incorporation of ecosystem services into decision-making, not only by governments, but also by businesses and individuals and by sectors such as agriculture, forestry, mining and land-use development planning (Pierce et al., 2005; Daily et al., 2009). This would require not only the consideration of conservation and management of natural land to protect and enhance ecosystem service provision, but also the consideration of the impacts of development objectives on the same.

Davids et al. (2016) identified the need for the consideration of ecosystem service areas within the municipal land-use decision-making frameworks through a possible independent conservation strategy for ecosystem services in Durban. This strategy would include the consideration of the large proportion of ecosystem service provisioning areas, lying outside of protected and managed areas, that are under threat of transformation.

With respect to planning for development within Durban, a proactive approach may be required in order to mitigate the impacts of development proposals within important ecosystem service areas. Observations and expert judgement suggest that the fact that no development applications were made in areas with a hotspot richness of five or more, could be attributed to the potential inaccessibility of these areas in terms of slope or the fact that these are located within drainage lines or rivers. These two factors may thus be considered as natural measures of protection for ecosystem services, except for sand mining activities, that generally target rivers and drainage lines.

The potential loss of important ecosystem service areas needs to be carefully considered prior to the implementation of planning and development proposals in Durban. Practical short-term governance solutions would be to include ecosystem service hotspot areas into D’MOSS where they are currently excluded, to develop guidelines for use of ecosystem service hotspots areas and to develop decision-making guidelines for development applications that fall within ecosystem service priority areas.

### Potential for future research

This research could be expanded in numerous ways. Firstly, the quantification of ecosystem services for various development scenarios would serve as a far more effective tool for planning future development. This could include quantifying the contribution of cultivated areas to ecosystem services provision, which was not assessed in this study. A spatial analysis to quantify accessibility to areas with high ecosystem service richness (referred to above), could confirm whether natural measures of protection exist for ecosystem service hotspots. The addition of cultural services or non-material NCP, would also provide a more comprehensive understanding of the importance of natural capital for the population of Durban and would provide further motivation for the protection of the same.

More work is required to assess the compatibility of various land uses to ecosystem service provision (Reyers et al., 2009), including research on the ecological processes linked to ecosystem services and how these are affected by land use change and the social and economic factors that drive changes (Fu et al., 2015). This will provide increased understanding of the links between social, economic and ecological factors that could prove more useful for decision-making.

## Conclusion

The study highlights that in order to achieve long term sustainability, there is a need to balance the demands of the increasing urbanization in Durban with the demands for human well-being that can be supported by ecosystems services. We highlight the potential societal impacts of strategic and local development threats to the five ecosystem service hotspot areas within city and confirm that management and governance responses are needed. The spatial consideration of threats to ecosystem services is an important tool to assist government to adequately plan for more sustainable cities, whereby development does not unduly impact on natural capital and its ability to provide critical contributions to people.

##  Supplemental Information

10.7717/peerj.5723/supp-1 Data S1 Raw numbers used in the analyses

10.7717/peerj.5723/supp-2 Appendix S1 Percentage of each proposed land use per hotspot in the North

10.7717/peerj.5723/supp-3 Appendix S2 Percentage of each proposed land use per hotspot in the Outer West

10.7717/peerj.5723/supp-4 Appendix S3 Distribution of function hotspots within landuses in the Outer West

10.7717/peerj.5723/supp-5 Appendix S4 Distribution of function hotspots within land uses in the North

## Additional Information and Declarations

### Data Availability

The following information was supplied regarding data availability: The raw data are provided in the tables and figures in the article.
