# Supplementary material for: Spatial analyses of threats to ecosystem service hotspots in Greater Durban, South Africa
Source: PeerJ. 2018 Oct 26;6:e5723. doi: 10.7717/peerj.5723 (PMC6204817; doi:10.7717/peerj.5723)
Supplement: Appendix S1 [file peerj-06-5723-s002.pdf]

SUPPLEMENTARY MATERIAL

Appendix 1

Percentage of each proposed land use per hotspot in the North Planning Region of the EMA

	Industry, Extractive, Airport, Tradeport	Commercial, Office Park, Mixed-use	Urban Residential	Rural Residential	Agriculture	Dam	Environment (D'MOSS, Public Open Space)
Carbon	2,59	2,42	16,75	3,70	12,05	0,07	62,42
Water yield	1,42	0,44	12,17	23,50	25,61	2,75	34,11
Flood attenuation Population	0,21	0,73	20,78	4,51	18,15	0,00	55,61
Flood attenuation public infrastructure	0,86	4,41	18,68	5,79	9,01	0,00	61,25
Flood attenuation private infrastructure	1,17	3,58	18,48	6,24	9,08	0,00	61,45
Sediment retention - dams	0,00	0,04	7,07	21,78	30,52	2,21	38,38
Sediment retention - sewer pipes	5,53	5,05	40,37	0,00	3,76	0,00	45,29
Sediment retention - storm drains	2,96	0,39	39,77	2,45	1,03	0,13	53,28
Sediment retention - harbour	0,00	0,00	0,00	0,00	0,00	0,00	0,00
Nitrogen retention - dams	0,00	0,98	21,57	32,35	4,90	0,98	39,22
Nitrogen retention - estuaries	0,00	0,00	38,10	11,11	0,00	0,00	50,79
Phosphorus retention - dams	0,00	0,00	5,00	20,00	0,00	0,00	75,00
Phosphorus retention - estuaries	0,00	0,00	5,00	20,00	0,00	0,00	75,00
**Average**	**1,23**	**1,50**	**20,31**	**12,62**	**9,51**	**0,51**	**54,32**
