# Supplementary material for: Spatial analyses of threats to ecosystem service hotspots in Greater Durban, South Africa
Source: PeerJ. 2018 Oct 26;6:e5723. doi: 10.7717/peerj.5723 (PMC6204817; doi:10.7717/peerj.5723)
Supplement: Appendix S2 [file peerj-06-5723-s003.pdf]

SUPPLEMENTARY MATERIAL

Appendix 2

Percentage of each proposed land use per hotspot in the Outer West Planning Region of the EMA

	Industry (Gen, Light, Wet)	Mixed use	Urban Residential, Cemetery, Landfill	Rural Residential, Tourism, Equestrian	Agriculture	Dam	Environment, Amenity
**Carbon**	1,16	0,83	3,68	11,67	0,80	0,09	81,76
**Water yield**	0,27	1,46	14,43	51,05	1,29	1,23	30,26
**Flood attenuation Pop**	0,00	0,22	4,32	24,56	0,00	0,00	70,90
**Flood attenuation public infrastructure**	0,00	0,05	4,51	18,27	0,00	0,10	77,06
**Flood attenuation private infrastructure**	0,00	1,14	4,07	18,04	0,00	0,09	76,65
**Sediment retention - dams**	1,16	0,53	6,33	31,81	1,27	0,19	58,71
**Sediment retention - sewer pipes**	1,71	1,48	15,80	7,76	0,18	0,00	73,06
**Sediment retention - storm drains**	0,84	1,01	13,83	14,72	0,04	0,00	69,55
**Sediment retention harbour**	0,00	0,64	6,94	18,33	0,00	0,00	74,09
**Nitrogen retention - dams**	0,42	0,00	3,81	47,15	0,00	0,21	48,41
**Nitrogen retention - estuaries**	0,00	0,00	7,34	42,20	0,00	0,00	50,46
**Phosphorus retention - dams**	2,00	0,67	7,33	31,33	0,00	0,67	58,00
**Phosphorus retention - estuaries**	0,00	1,16	32,56	27,91	0,00	1,16	37,21
**Average**	0,58	0,71	9,61	26,52	0,28	0,29	62,01
