# Supplementary material for: Spatial analyses of threats to ecosystem service hotspots in Greater Durban, South Africa
Source: PeerJ. 2018 Oct 26;6:e5723. doi: 10.7717/peerj.5723 (PMC6204817; doi:10.7717/peerj.5723)
Supplement: Appendix S3 [file peerj-06-5723-s004.pdf]

## SUPPLEMENTARY MATERIAL

### Appendix 3

**Distribution of function hotspots within landuses in the Outer West Planning Region**

| **Outer-west SDP land-uses** | Carbon | Water yield | Sediment retention (all) | Nutrient retention (all) | Flood attenuation (all) | Average Totals |
|---|---|---|---|---|---|---|
| **Industry (Gen, Light, Wet)** | 1.50 | 0.63 | 1.29 | 0.81 | 0.00 | 0.84 |
| **Mixed use / Town Centre / Business Park** | 0.84 | 2.04 | 0.80 | 0.32 | 0.76 | 0.95 |
| **Urban residential, Cemetery, Landfill** | 3.71 | 6.61 | 8.82 | 7.61 | 5.07 | 6.37 |
| **Agriculture, Equestrian** | 1.18 | 5.82 | 1.39 | 0.00 | 0.21 | 1.72 |
| **Dam** | 0.11 | 2.87 | 0.17 | 0.49 | 0.12 | 0.75 |
| **Rural Residential / Tourism** | 6.51 | 46.61 | 25.48 | 43.53 | 11.88 | 26.80 |
| **Environment, green corridor, amenity** | 86.15 | 35.42 | 62.04 | 47.25 | 81.95 | 62.56 |
