# Supplementary material for: Spatial analyses of threats to ecosystem service hotspots in Greater Durban, South Africa
Source: PeerJ. 2018 Oct 26;6:e5723. doi: 10.7717/peerj.5723 (PMC6204817; doi:10.7717/peerj.5723)
Supplement: Appendix S4 [file peerj-06-5723-s005.pdf]

SUPPLEMENTARY MATERIAL

Appendix 4

Distribution of function hotspots within land uses in the North Planning Region

Northern SDP Land uses	Carbon	Water yield	Sediment retention (all)	Nutrient retention (all)	Flood attenuation (all)	Average Total
**Industry / Extractive / Airport / Tradeport**	3.14	1.88	2.54	3.50	1.53	2.52
**Commercial/Office Park/mixed-use**	1.53	0.00	2.38	0.00	2.33	1.25
**Urban residential**	16.28	2.92	20.17	37.76	20.71	19.57
**Agriculture**	14.89	34.36	24.16	3.50	14.46	18.27
**Dam**	0.09	3.69	1.64	0.70	0.00	1.22
**Rural Residential**	4.50	31.23	16.28	32.87	6.40	18.25
**Environment (D'MOSS / POS)**	59.58	25.92	32.84	21.68	54.58	38.92
